# Muscle MR Radiomics for Evaluation of Idiopathic Inflammatory Myopathies

**DOI:** 10.2174/0115734056428840260211024728

**Published:** 2026-03-12

**Authors:** Wenyan Zhang, Qin Li, Gang Wu

**Affiliations:** 1 Department of Radiology, Tongji Hospital, Tongji Medical College, Huazhong University of Science and Technology, Wuhan, China; 2 Department of Nursing, Tongji Hospital, Tongji Medical College, Huazhong University of Science and Technology, Wuhan, China

**Keywords:** Idiopathic inflammatory myopathies (IIM), Muscle MR radiomics, Predication model, Least absolute shrinkage and selection operator (LASSO) regression, Invasive muscle biopsy

## Abstract

**Purpose:**

This study aimed to establish a prediction model for Idiopathic Inflammatory Myopathies (IIM) based on the radiomics of vastus intermedius.

**Methods:**

43 IIM patients and 48 control cases were analyzed in the retrospective study. By using the 3D slicer software, 107 radiomics features were obtained for each case. The selection of variables was performed using a two-sample t-test or the Mann-Whitney U test, followed by maximum correlation minimum redundancy (mRMR). Least Absolute Shrinkage and Selection Operator (LASSO) regression was used for dimension reduction. To assess the model performance, the receiver operating characteristic (ROC) curve was plotted.

**Results:**

The results showed that 67 features were varying between the IIM cases and control groups (*p* < 0.05). With the mRMR and LASSO methods, seven features were finally determined. Regarding the radiomics model's performance, it achieved an AUC of 0.983 in the validation data.

**Discussion:**

This study investigated microstructural differences in a specific muscle (varasimus intermedias) between Idiopathic Inflammatory Myopathy (IIM) patients and normal controls using radiomics. It identified 67 distinguishing radiomics features and successfully established a high-performance prediction model (AUC >0.9) using LASSO and mRMR for feature selection. These features likely reflect IIM-induced microstructural changes, such as inflammation and necrosis, typically visible on MRI as signal abnormalities. Radiomics thus offers a potential non-invasive alternative to muscle biopsy for assessing these changes.

**Conclusion:**

Muscle MR radiomics is feasible for identifying idiopathic inflammatory myopathy and has the potential to serve as a non-invasive alternative to muscle biopsy.

## 1 INTRODUCTION

Idiopathic inflammatory myopathies (IIM) are a heterogeneous group of autoimmune-mediated systemic connective tissue diseases [1]. According to current data, the incidenceofIIMisreportedto bebetween 0.2 and 2per 100,000 person - years, and the prevalence is documented as ranging from 2 to 25 per 100,000 individuals [2]. The primary pathological characteristics involve the infiltration of skeletal muscle by inflammatory cells as well as the necrosis, degeneration, and regeneration of muscle fibers. Clinically, patients mainly present with symmetrical weakness of the proximal limb muscles, limb girdle muscles, neck muscles, and throat muscles. The condition typically involves multiple organs and can be accompanied by other connective tissue diseases. The types of myositis include polymyositis (PM), dermatomyositis (DM), and inclusion body myositis (IBM) [3, 4, 5]. IIM often starts from the lower limbs, which are manifested as difficulties in climbing up and down stairs and stepping up and down, while the upper limbs are involved in laborious lifting. Half of the patients with neck muscle involvement, difficulty in raising their head in the lying position or weakness in the sitting position were observed. Muscle pain is seen in about one-third of patients. Upon damage to muscle fibers, numerous muscle enzymes can be released into the blood, causing a notable rise in serum muscle enzyme levels. Creatine kinase (CK), especially, may show an increase of several - fold to several tens of times [6, 7], which can reflect the severity of muscle damage in the early and middle stages of inflammatory myopathy, or indicate that the disease is in an active stage. In the diagnosis of idiopathic inflammatory myopathies (IIM), antinuclear antibody (ANA) is the most frequently encountered autoantibody. It exhibits a high positivity rate among IIM patients, particularly in those with dermatomyositis and polymyositis, where the positivity rate of ANA can reach approximately 80%. One -third of patients have autoantibodies with diagnostic significance, which are called myositis-specific antibodies (MSA) [8]. Invasive muscle biopsy is the decisive diagnostic method, and MRI is very useful in initial diagnosis and dynamic monitoring [9]. Diffuse hyperintensity on fat-suppressed T2-weighted image is the main finding for IIM cases [10].

CT or MR imaging radiomics has been successfully utilized in the diagnosis and assessment of various muscle diseases, demonstrating promising outcomes [11, 12]. However, to the best of the author’s knowledge, there is no publication regarding the comparison of the MR radiomics features of IIM muscle and normal muscle. In the process of extracting radiomics features, ROI selection and drawing are much more difficult for IIM cases than other MSK disease cases, such as soft tissue tumor cases. In this study, we decided to limit our investigation to the the vastus intermedius of the thigh. The vastus intermedius muscle was selected for investigation primarily due to its relatively regular and representative anatomical morphology compared to other thigh muscles in the context of this study. We aimed to establish a prediction model based on the radiomics of the vastus intermedius.

## 2 MATERIALS AND METHODS

### 2.1 Study Design and Participants

This is a retrospective case-control study approved by the institutional ethics committee (approval number: TJ-IRB202501020). A retrospective cohort analysis was conducted of patients meeting pre-defined eligibility criteria at our institution between January, 2004 and March, 2024. Inclusion criteria of the case group (IIM patients): clinical diagnosis of idiopathic inflammatory myopathy (IIM); availability of axial T2-weighted MRI of the thigh. Exclusion criteria: suboptimal image quality; presence of internal metallic implants; slice thickness >6 mm; muscle biopsy performed within 48 hours preceding MRI. Inclusion criteria of the control group (non-IIM): exclusion of IIM diagnosis; availability of axial T2-weighted MRI of the thigh. Exclusion criteria: suboptimal image quality; presence of internal metallic implants; slice thickness >6 mm; rheumatoid arthritis; acute traumatic thigh injury (<6 weeks); soft tissue tumors; active soft tissue infection; peripheral neuropathy affecting lower extremities. Clinical diagnosis of idiopathic inflammatory myopathy (IIM) was established using the 2017 EULAR/ACR classification criteria for adult and juvenile IIM [13]. The study followed the Sex and Gender Equity in Research guidelines (SAGER) to ensure appropriate inclusion and reporting of sex-related data.

### 2.2 Images Analysis

All dicom data from patients were transferred to a dedicated workstation equipped with a 16 GB GPU and subsequently converted to nii format using 3D Slicer software. A radiologist with 10 years of experience was requested to conduct three-dimensional segmentation of the vastus intermedius using 3D Slicer. Radiomics features of the vastus intermedius were extracted for all cases. When bilateral thighs were displayed on transverse images, only the right thigh was used for feature extraction. To address potential sources of bias, an associate professor of radiology performed a secondary inspection of all images and data. We did not perform feature extraction for other muscles of the thigh for simplicity. The following feature classes were selected: first-order, glcm, gldm, glrlm, glszm, ngtdm, shape, and shape 2D. A total of 107 features were extracted for each case in the study.

### 2.3 Statistical Analysis

In this study, the radiomics features data were used to build a prediction model for IIM, using Anaconda (Python 3.10). To compare differences between the case and control groups, we employed the two-sample t-test for data that followed a normal distribution and the Mann - Whitney U test for non - normal continuous variables. For features with p-values less than 0.05, they were incorporated into the analysis of Maximum Relevance and Minimum Redundancy(mRMR) and Least Absolute Shrinkage and Selection Operator (LASSO) regression. The training and validation cohorts were divided at a ratio of 3:1. Thus, cases were allocated to the training and validation cohorts. The receiver operating characteristic (ROC) curve was constructed to evaluate the model's performance, and the area under the curve (AUC) was calculated. Finally, the application value of the prediction model was evaluated by decision curve analysis (DCA).

## 3 RESULTS

**Fig. (1):**
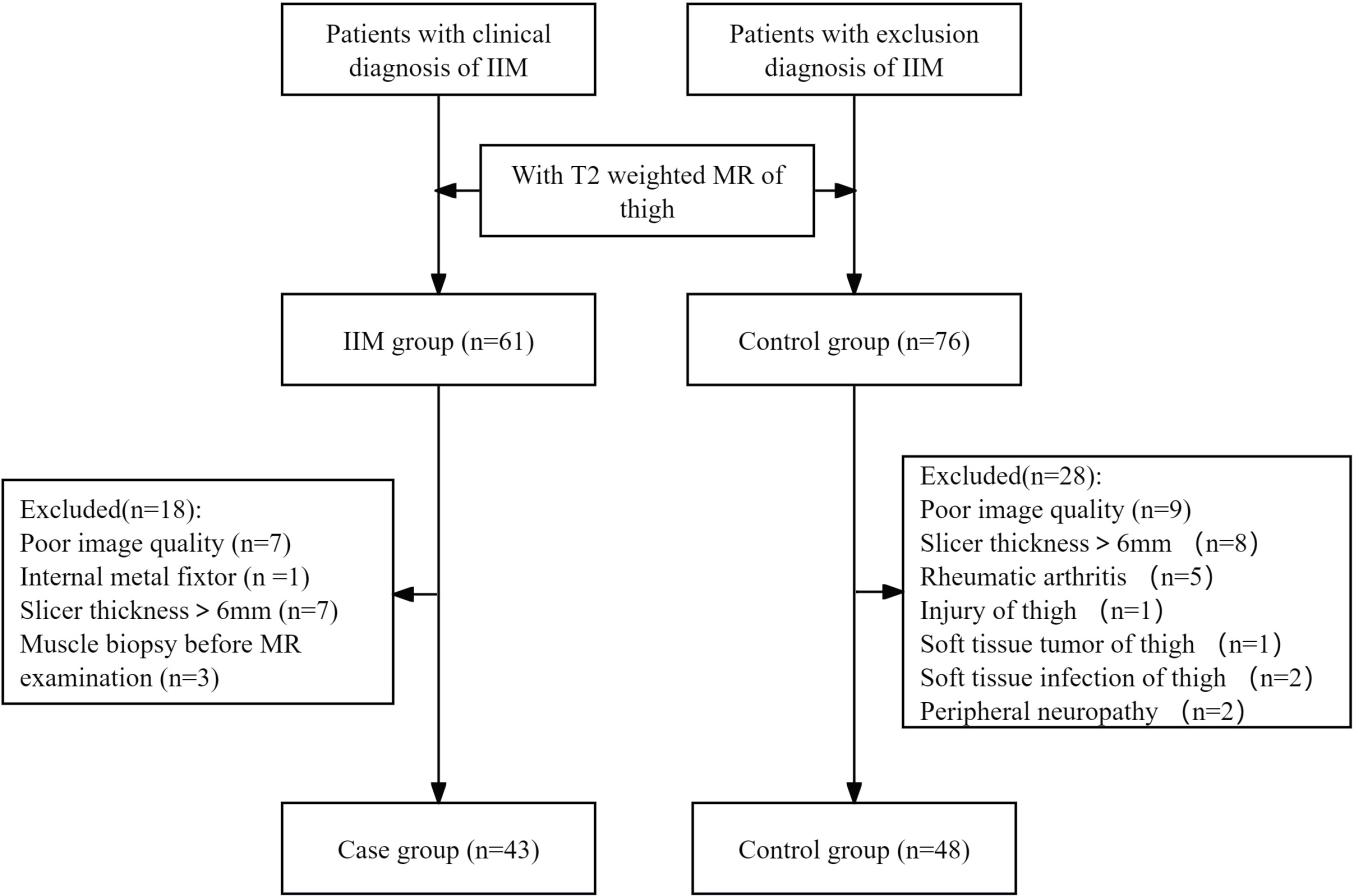
Flowchart of case screening process.

The study analyzed 43 patients in the case group and 48 patients in the control group between 2004 and 2024 (Fig. **1**). No missing data was observed. The patients' ages ranged from 13 to 77 years, with a median age of 55 years. The biological male to female ratio was 1.2. In the study, no significant differences were observed between the two groups in terms of age or gender (*p* > 0.05). However, the case and control groups exhibited differences in 67 features (*p* < 0.05).

**Fig. (2):**
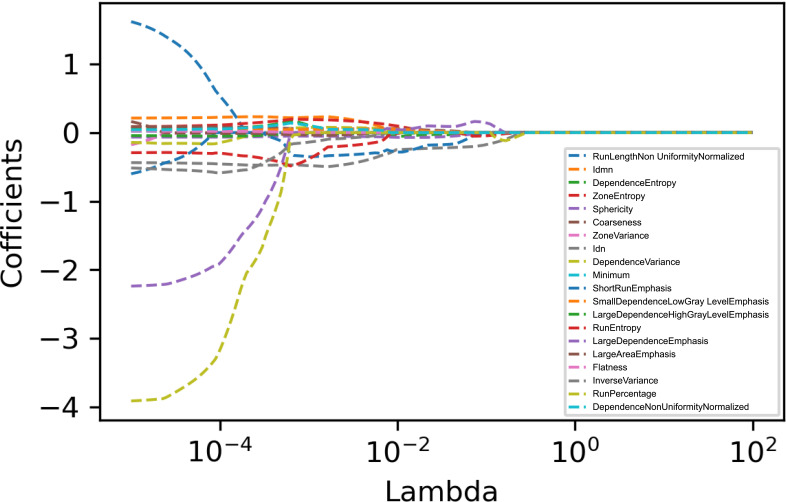
The LASSO coefficient profile plot illustrates how feature coefficients vary with lambda values during model construction, demonstrating the influence of regularization strength on feature selection.

Through the application of the mRMR method, 20 features were ultimately selected (Table **1**). The Lasso regression was employed to select the most relevant features for the predictive model (Fig. **2**). As shown in Fig. (**3**), the coefficients of the final selected seven features were estimated using the LASSO regression. Consistent with the '*10 Events Per Variable*' (EPV) principle for predictive modeling, our final cohort of 91 cases exceeded the minimum requirement of 70 cases needed to develop a stable model with seven predictor variables, thereby ensuring robust model development. The results indicated that LargeDependenceEmphasis and Idn were the most significant features, with the largest absolute values of coefficients. LargeDependenceEmphasis had a positive coefficient, suggesting a positive association with the outcome, while Idn had a negative coefficient, indicating a negative association. Other features, including Coarseness, SmallDependence-LowGrayLevelEmphasis, DependenceEntropy, Sphericity, and ShortRunEmphasis, had relatively smaller coefficients, implying that their contributions to the model were less prominent. The LASSO regression successfully identified the key features that drove the model's predictions, providing insights into the underlying factors associated with the outcome of interest.

**Fig. (4):**
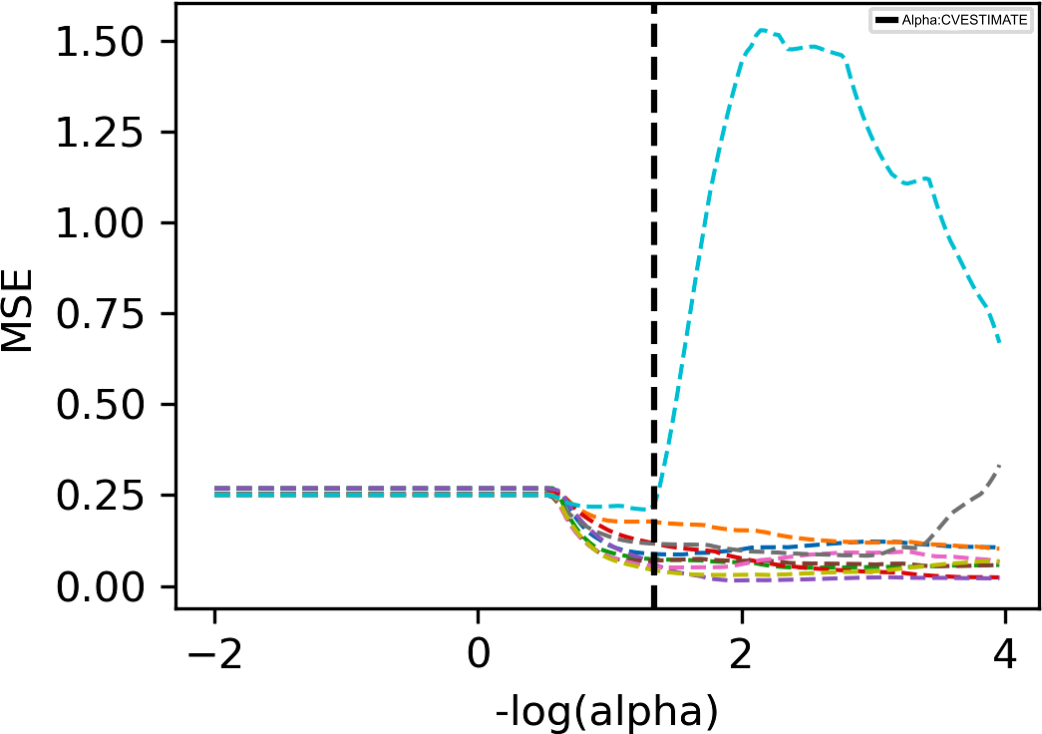
The trend of the mean square error (MSE) of the model as a function of the regularization parameter (alpha) was shown. **Abbreviations:** MSE, mean squared error.

Through ten-fold cross-validation, we selected the alpha value that minimized the MSE (dotted line marked). The model under this alpha value performed best on the validation set while maintaining good generalization ability (Fig. **4**).

**Fig. (5):**
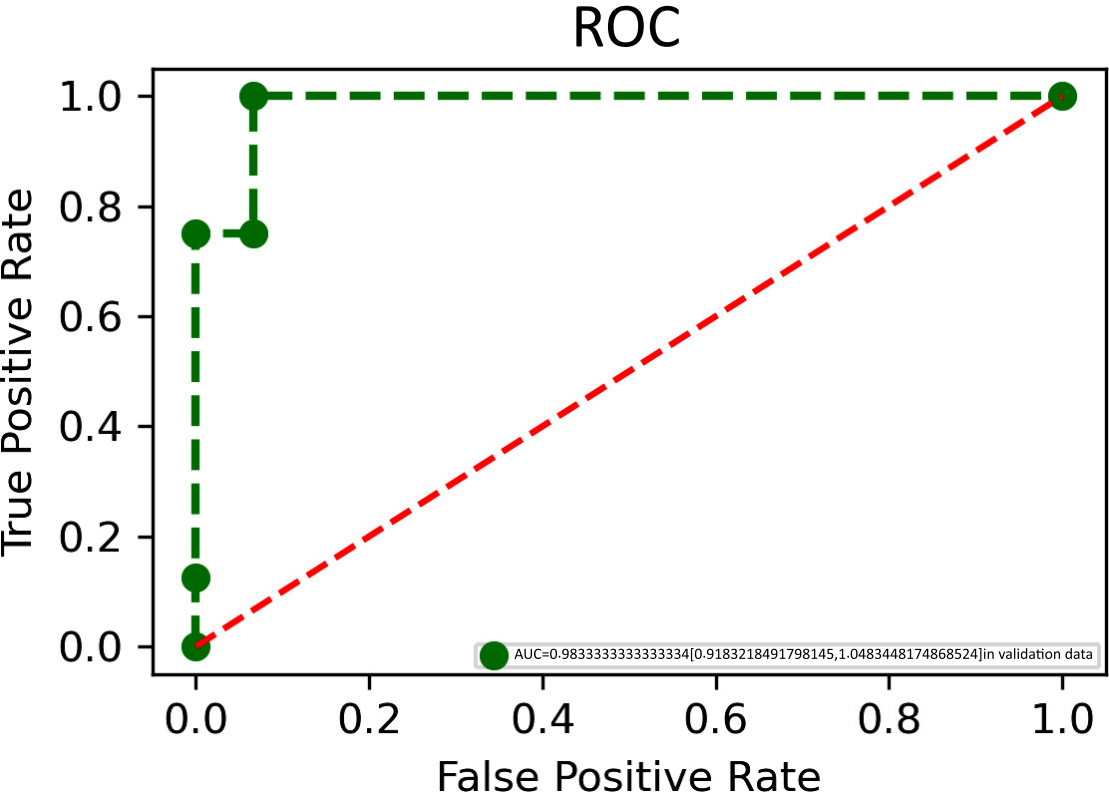
The ROC curve of the model. The performance of the radiomics model in the validation group was evaluated using the ROC curve, with an AUC of 0.983, indicating excellent discrimination capability. **Abbreviations:** ROC, receiver operating characteristic; AUC, area under the curve.

Based on radiomics features extracted from vastus intermedius datasets, the model demonstrated excellent diagnostic performance in the validation cohort: AUC = 0.983 (95% CI: 0.918–1.000), sensitivity = 0.977 (95% CI: 0.934–1.000), specificity = 0.938 (95% CI: 0.865–1.000), and accuracy = 0.956 (95% CI: 0.914–0.998) (Fig. **5**).

Radiomics score was calculated by the following formula: Radiomics score = -0.216377×Idn - 0.162847×ShortRun-Emphasis - 0.049000×Sphericity - 0.031495×Dependence-Entropy + 0.012612×Small-DependenceLowGrayLevel-Emphasis + 0.020977 ×Coarseness + 0.088746×LargeDepen-denceEmphasis.

The decision curve analysis (DCA) was performed to evaluate the clinical utility of the predictive model. As shown in Fig. (**6**), the model showed a higher net benefit than “Treat none” across the majority of threshold probabilities and outperformed “Treat all” in the threshold probability range of approximately 0.1 to 0.7. These findings indicated that the model has the potential to effectively identify individuals who are likely to benefit from intervention and could offer more value than simple treat-all or treat-none approaches. Overall, the DCA suggested that the model may serve as a useful tool for guiding clinical decision-making by providing personalized risk assessment.

## 4 DISCUSSION

The current study investigated whether IIM and normal cases differed in the microstructure of vastus intermedius and tried to establish a prediction model based on radiomics. Key findings included: firstly, 67 radiomics features distinguished vastus intermedius of IIM from normal cases, seven features were determined in the final model; secondly, the prediction model using vastus intermedius features demonstrated an AUC exceeding 0.9.

### 4.1 Pathological and Imaging Features of IIM

The pathological changes in the muscle tissue of IIM patients are primarily characterized by inflammatory infiltration, microcirculation hemorrhage, and extensive muscle fiber necrosis [14, 15]. Pinalfernandez *et al* performed MRI scanning on 666 patients with different types of myositis, including 219 patients with dermatomyositis, 176 patients with polymyositis, and 153 patients with inclusion body myositis [16]. The study showed that dermatomyositis patients had a higher frequency of fascial edema and fasciitis compared to other myositis types, immune-mediated necrotizing myopathy patients often had muscle edema, and inclusion body myositis patients had distinct, often asymmetrical muscle involvement compared to other myositis types. Although there are certain differences in the specific image manifestations of different types of myositis [13], MRI imaging shows diffuse or grid-like hyperintensities in muscle tissue or fascia between tissues, which are shown as long T1 and long T2 signals, suggesting extensive inflammatory infiltration, microcirculation hemorrhage, and massive necrosis of capillaries and muscle fibers in the muscle tissue of IIM patients [17]. Some patients with longer disease duration observe localized short T1 and slightly long T2 signals in muscle tissue, indicating fatty infiltration in muscle tissue, which is not found in the muscle tissue of obese normal people, suggesting massive atrophy of muscle fibers in muscle tissue and replacement by adipose tissue.

Invasive muscle biopsy is the best method to study the tissue's microscopic changes of inflammatory myopathy. CT or MRI imaging omics has also been reported to reveal the microscopic changes of skeletal muscle tissue, so it can be used as a noninvasive alternative to skeletal muscle biopsy [18, 19]. In the study, we found IIM cases and normal cases differed in more than sixty features of vastus intermedius. The most possible explanation is that the microstructure of vastus intermedius has been changed by IIM.

### 4.2 Performance of the Model

LASSO and mRMR are the main dimension reduction methods used in this study. The principle for mRMR is very simple. The primary objective is to identify a subset of features from the original feature set that demonstrate maximum relevance to the final output result while exhibiting minimal redundancy among themselves [20]. LASSO can cause parameter sparsity because the norm penalty can shrink some coefficients to zero, thus realizing feature selection. The coefficients corresponding to non-important features are completely set to zero. LASSO can reduce overfitting, because the regularization term can prevent the model from overfitting on the training data, thus improving the generalization ability [21]. By selecting a small number of important features, LASSO can generate simpler and more interpretable models. In the study, *via* the application of LASSO and mRMR, a select few features were chosen to construct a prediction model. This model extraordinarily performed an AUC exceeding 0.9, which is substantially higher than that achievable by any single feature.

### 4.3 Limitations

The study has several limitations. Firstly, the sample size was relatively small. Since IIM is a low-incidence disease, only 43 cases were collected over many years. Secondly, a muscle biopsy wasn't used as the reference standard; the IIM diagnosis was solely based on clinical assessment. Thirdly, the radiomics method wasn't compared with other computer vision techniques. Future studies should focus on gathering more cases through multi-center collaborations to enhance model robustness and generalizability. Despite the challenges, efforts should be made to incorporate muscle biopsy as a reference standard to improve diagnostic accuracy. Furthermore, future research should include comparisons between the radiomics method and other advanced computer vision techniques, such as convolutional neural networks.

## CONCLUSION

This study successfully developed and validated a radiomics model based on T2-weighted MR images of the vastus intermedius for the identification of idiopathic inflammatory myopathies (IIM). The model, constructed using seven selected features following mRMR and LASSO regression, demonstrated excellent diagnostic performance with an AUC of 0.983 in the validation cohort, along with high sensitivity and specificity. These findings suggest that radiomics analysis can non-invasively detect microstructural alterations in muscle tissue resulting from IIM, supporting its potential as a valuable adjunct to current diagnostic workflows. Despite limitations related to sample size and the clinical reference standard, this work establishes muscle MR radiomics as a promising, non-invasive tool that may reduce reliance on muscle biopsy in select clinical scenarios. Future studies with larger, multi-center cohorts and histopathological correlation are warranted to extensive validation and refining of this approach.

## Figures and Tables

**Fig. (3):**
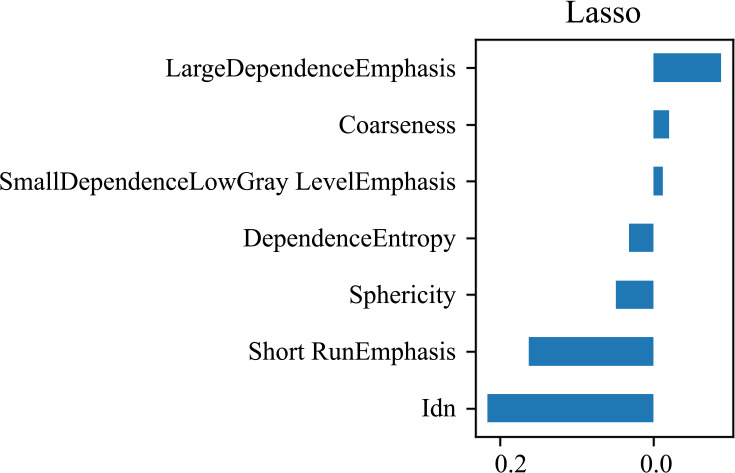
The LASSO bar plot demonstrates the selected features and their corresponding regression coefficients in the model, highlighting the most significant features contributing to the prediction. **Abbreviations:** LASSO, Least Absolute Shrinkage and Selection Operator.

**Fig. (6):**
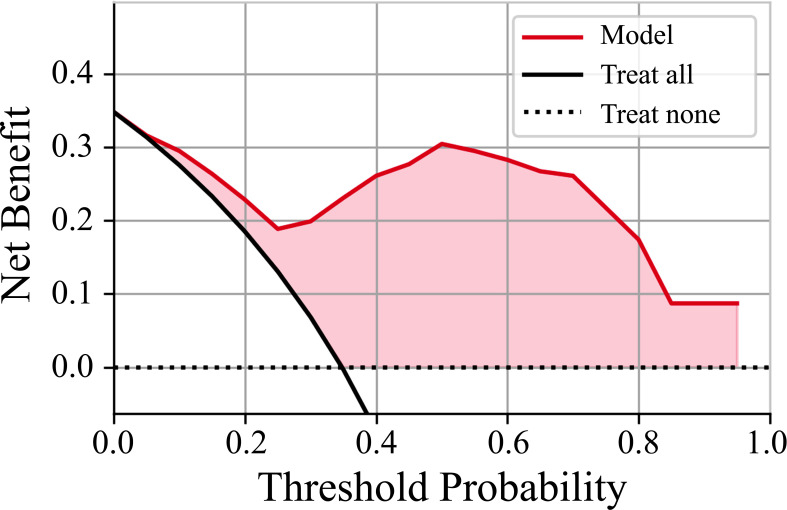
Decision Curve Analysis (DCA) of the predictive model. The net benefit of the model (red line) is plotted against the threshold probability. Compared with the “Treat all” (black line) and “Treat none” (dotted black line) strategies, the model provides greater net benefit across a range of threshold probabilities. **Abbreviations:** DCA, decision curve analysis.

**Table 1:** The details of features selected by mRMR, including the coefficient in the LASSO formula and accuracy in identifying disease.

**Variable**	**LASSO coefficient**	**AUC(95%CI)**	**Sensibility(95%CI)**	**Specificity(95%CI)**	**Accuracy(95%CI)**
RunLengthNonUniformityNormalized	0	0.8(0.71,0.89)	0(0,0)	1(1,1)	0.47(0.37,0.58)
Idmn	0	0.79(0.69,0.88)	0(0,0)	1(1,1)	0.47(0.37,0.58)
DependenceEntropy	-0.031495	0.77(0.67,0.86)	0(0,0)	1(1,1)	0.47(0.37,0.58)
ZoneEntropy	0	0.69(0.58,0.8)	0(0,0)	1(1,1)	0.47(0.37,0.58)
Sphericity	-0.049	0.63(0.52,0.75)	0(0,0)	1(1,1)	0.47(0.37,0.58)
Coarseness	0.020977	0.73(0.63,0.84)	0.74(0.62,0.87)	0.69(0.55,0.83)	0.73(0.63,0.82)
ZoneVariance	0	0.72(0.62,0.83)	0.72(0.59,0.85)	0.69(0.55,0.83)	0.71(0.62,0.81)
Idn	-0.216377	0.8(0.7,0.89)	0.02(0,0.07)	0.98(0.94,1.02)	0.47(0.37,0.58)
DependenceVariance	0	0.8(0.7,0.89)	0.72(0.59,0.85)	0.79(0.67,0.91)	0.76(0.67,0.85)
Minimum	0	0.56(0.44,0.68)	0(0,0)	1(1,1)	0.47(0.37,0.58)
ShortRunEmphasis	-0.162847	0.8(0.71,0.9)	0(0,0)	1(1,1)	0.47(0.37,0.58)
SmallDependenceLowGrayLevelEmphasis	0.012612	0.76(0.66,0.86)	0.84(0.73,0.94)	0.58(0.44,0.73)	0.71(0.62,0.81)
LargeDependenceHighGrayLevelEmphasis	0	0.8(0.71,0.89)	0.95(0.89,1.01)	0.06(0,0.13)	0.54(0.44,0.64)
RunEntropy	0	0.8(0.71,0.89)	0(0,0)	1(1,1)	0.47(0.37,0.58)
LargeDependenceEmphasis	0.088746	0.8(0.71,0.89)	0.67(0.54,0.81)	0.88(0.78,0.97)	0.77(0.68,0.86)
LargeAreaEmphasis	0	0.72(0.62,0.83)	0.72(0.59,0.85)	0.69(0.55,0.83)	0.71(0.62,0.81)
Flatness	0	0.65(0.54,0.76)	0.02(0,0.07)	1(1,1)	0.48(0.38,0.59)
InverseVariance	0	0.63(0.52,0.75)	0.93(0.86,1)	0.42(0.27,0.56)	0.69(0.6,0.79)
RunPercentage	0	0.8(0.71,0.9)	0(0,0)	1(1,1)	0.47(0.37,0.58)
DependenceNonUniformityNormalized	0	0.79(0.7,0.88)	0(0,0)	1(1,1)	0.47(0.37,0.58)

## Data Availability

The data of current study are available from corresponding author, [G.W.], on a reasonable request.

## LIST OF ABBREVIATIONS

IIM: Idiopathic Inflammatory Myopathies
PM: Polymyositis
DM: Dermatomyositis
IBM: Inclusion Body Myositis
CK: Creatine Kinase
ANA: Antinuclear Antibody
MSA: Myositis Specific Antibodies
mRMR: Maximum Relevance and Minimum Redundancy
LASSO: Least Absolute Shrinkage and Selection Operator
ROC: Receiver Operating Characteristic
AUC: Area Under the Curve

## STANDARDS OF REPORTING

STROBE guidelines were followed

## References

[1] Dalakas M.C. (2015). Inflammatory muscle diseases.. N Engl J Med..

[2] Khoo T., Lilleker J.B., Thong B.Y.H., Leclair V., Lamb J.A., Chinoy H. (2023). Epidemiology of the idiopathic inflammatory myopathies.. Nat. Rev. Rheumatol..

[3] Damoiseaux J., Vulsteke J.B., Tseng C.W., Platteel A.C.M., Piette Y., Shovman O., Bonroy C., Hamann D., De Langhe E., Musset L., Chen Y.H., Shoenfeld Y., Allenbach Y., Bossuyt X. (2019). Autoantibodies in idiopathic inflammatory myopathies: Clinical associations and laboratory evaluation by mono- and multispecific immunoassays.. Autoimmun. Rev..

[4] Lundberg I.E., Fujimoto M., Vencovsky J., Aggarwal R., Holmqvist M., Christopher-Stine L., Mammen A.L., Miller F.W. (2021). Idiopathic inflammatory myopathies.. Nat. Rev. Dis. Primers.

[5] Wu Y., Luo J., Duan L. (2024). Pathogenic mechanisms of disease in idiopathic inflammatory myopathies: Autoantibodies as clues.. Front. Immunol..

[6] Ashton C., Paramalingam S., Stevenson B., Brusch A., Needham M. (2021). Idiopathic inflammatory myopathies: A review.. Intern. Med. J..

[7] Zhang L., Zhu H., Yang P., Duan X., Wei W., Wu Z., Fang Y., Li Q., Liu S., Shi X., Li H., Wu C., Zhou S., Leng X., Zhao J., Xu D., Wu Q., Tian X., Li M., Zhao Y., Wang Q., Zeng X. (2021). Myocardial involvement in idiopathic inflammatory myopathies: A multi-center cross-sectional study in the CRDC-MYO Registry.. Clin. Rheumatol..

[8] Zhu L., Zong C., Chen Y., Wang G., Ge Y. (2024). Clinical characteristics of idiopathic inflammatory myopathies patients with anti-PM/Scl antibodies.. Semin. Arthritis Rheum..

[9] Malartre S., Bachasson D., Mercy G., Sarkis E., Anquetil C., Benveniste O., Allenbach Y. (2021). MRI and muscle imaging for idiopathic inflammatory myopathies.. Brain Pathol..

[10] Takei H., Kondo Y., Takanashi S., Takeuchi T., Matsubara S., Kaneko Y. (2025). Contrast of muscle magnetic resonance imaging and pathological findings of muscle tissue in patients with anti-aminoacyl transfer RNA synthetase antibodies.. Mod. Rheumatol..

[11] Guo J., Meng W., Li Q., Zheng Y., Yin H., Liu Y., Zhao S., Ma J. (2024). Pretreatment sarcopenia and MRI-based radiomics to predict the response of neoadjuvant chemotherapy in triple-negative breast cancer.. Bioengineering.

[12] Hu M., Zheng F., Ma X., Liu L., Shen C., Wu J., Wang C., Yang L., Xu Y., Zou L., Fei L., Lu M., Xu X. (2022). Assessment of thigh MRI radiomics and clinical characteristics for assisting in discrimination of juvenile dermatomyositis.. J. Clin. Med..

[13] Lundberg IE., Tjärnlund  A., Bottai M. (2017). 2017 European League Against Rheumatism/American College of Rheumatology classification criteria for adult and juvenile idiopathic inflammatory myopathies and their major subgroups.. Ann. Rheum. Dis..

[14] Aouizerate J., De Antonio M., Bader-Meunier B. (2018). Muscle ischaemia associated with NXP2 autoantibodies: A severe subtype of juvenile dermatomyositis.. Rheumatology.

[15] Suárez-Calvet X., Gallardo E., Pinal-Fernandez I. (2017). RIG-Iexpression in perifascicular myofibers is a reliable biomarker of dermatomyositis.. Arthritis Res Ther.

[16] Pinal-Fernandez I, Casal-Dominguez M., Carrino JA. (2017). Thigh muscle MRI in immune-mediated necrotising myopathy: Extensive oedema, early muscle damage and role of anti-SRP autoantibodies as a marker of severity.. Ann. Rheum. Dis..

[17] Paramalingam S., Counsel P., Mastaglia F.L., Keen H., Needham M. (2019). Imaging in the diagnosis of idiopathic inflammatory myopathies; indications and utility.. Expert Rev. Neurother..

[18] Huang Z.G., Gao B.X., Chen H., Yang M.X., Chen X.L., Yan R., Lu X., Shi K.N., Chan Q., Wang G.C. (2017). An efficacy analysis of whole-body magnetic resonance imaging in the diagnosis and follow-up of polymyositis and dermatomyositis.. PLoS One..

[19] Pipitone N. (2016). Value of MRI in diagnostics and evaluation of myositis.. Curr. Opin. Rheumatol..

[20] Ren Z., Ren G., Wu D. (2022). Deep learning based feature selection algorithm for small targets based on mRMR.. Micromachines.

[21] Das P., Babadi B. (2023). Non-asymptotic guarantees for reliable identification of granger causality *via* the LASSO.. IEEE Trans. Inf. Theory.

